# Production, Transport, and Metabolism of Volatile Fatty Acids in the Yak Rumen: Unraveling the Unique Mechanisms Underpinning High-Altitude Adaptation

**DOI:** 10.3390/microorganisms14030696

**Published:** 2026-03-19

**Authors:** Zhenyu Zhu, Jianbo Zhang, Ali Mujtaba Shah, Qunying Zhang, Binqiang Bai, Lizhuang Hao

**Affiliations:** 1 Key Laboratory of Plateau Grazing Animal Nutrition and Feed Science of Qinghai Province, Qinghai University, Xining 810016, China; mrzhu2024@outlook.com (Z.Z.);; 2 State Key Laboratory of Swine and Poultry Breeding Industry, College of Animal Science, South China Agricultural University, Guangzhou 510642, China

**Keywords:** *Bos grunniens*, rumen microbiota, high-altitude adaptation, rumen epithelium, energy metabolism, short-chain fatty acids

## Abstract

Volatile fatty acids (VFAs), the primary end-products of microbial fermentation in the ruminant forestomach, supply approximately 70% of the host’s energy requirements and play a pivotal role in maintaining energy homeostasis. While the mechanisms governing ruminal VFA production, absorption, and metabolism are well-characterized in common ruminants like dairy and beef cattle, a systematic integration of these processes in yaks, an iconic species long-adapted to the extreme Qinghai–Tibet Plateau, remains incomplete. This review synthesizes current knowledge on the entire VFA pathway in the yak rumen, from production to tissue metabolism. We detail the critical roles of functional microbes, including fibrolytic bacteria and Prevotella, in VFA synthesis and how their activity is dynamically regulated by dietary composition and seasonal shifts. Building on the unique structural features of the yak rumen epithelium, the review analyzes VFA absorption mechanisms involving both passive diffusion and carrier-mediated transport. Furthermore, we systematically outline the metabolic fates and energy partitioning strategies of VFAs across the rumen epithelium, liver, and peripheral tissues. This synthesis aims to elucidate the highly efficient and adaptive physiological basis of VFA metabolism that underpins the yak’s exceptional ability to utilize energy under the low-energy conditions of the high-altitude environment. Ultimately, this work seeks to provide a theoretical foundation for understanding plateau-adapted energy efficiency and to inform precision nutritional strategies for ruminants in alpine regions.

## 1. Introduction

The rumen functions as a highly efficient natural bioreactor. VFAs (primarily acetate, propionate, and butyrate) generated through microbial metabolism are central to host energy homeostasis, meeting roughly 70% of a ruminant’s energy demand, and are often regarded as the “energy currency” driving growth, development, and production [1]. VFA dynamics encompass a complex biological process involving microbial community interplay, epithelial transport, and multi-tissue metabolic integration [2]. Advances in multi-omics and in vitro techniques have led to systematic elucidation of VFA metabolism in the rumen of conventional ruminants, spanning rumen microecology, host nutrient absorption [3,4], and nutritional intervention [5,6,7]. For yaks (*Bos grunniens*), however, millennia of natural selection and domestication on the high-altitude plateau have fostered distinct rumen morphophysiology, microbial ecosystems, and host metabolic networks, collectively forming an optimized system for energy conservation and acquisition [8,9]. Despite this, a comprehensive review integrating the complete pathway of ruminal VFA production, transport, and metabolism in yaks is still lacking, particularly regarding intrinsic regulatory responses to extreme seasonal environmental fluctuations.

These distinctive adaptations of the yak are fundamentally rooted in the structure and function of its rumen. Through symbiotic fermentation with a diverse consortium of microbes, including bacteria, archaea, protozoa, and fungi, the host converts indigestible plant material into essential nutrients [2]. This fermentative efficiency is further supported by a suite of morphological and microbial specializations. The yak’s specialized tongue structure enhances physical digestion [10], while its rumen epithelium features high papillae density and an expanded absorptive surface area [11,12], providing a morphological foundation for efficient VFA uptake, which is crucial for rapid energy acquisition in cold climates. Morphological traits like short, stout limbs and a dense fur coat further minimize energy loss and optimize energy allocation [8]. Microbial community analyses reveal significant spatial heterogeneity along the yak gastrointestinal tract. The rumen and abomasum harbor similar microbial community structures, a pattern that likely reflects the transit of rumen-derived microbes to the abomasum rather than equivalent functional activity of the microbiota in the abomasum, and the microbial compositions of both digestive compartments are distinct from that of the jejunum. Notably, acetate and butyrate concentrations in the rumen show a strong positive correlation with ruminal microbial community composition [13]. Comparative studies across altitudes indicate an enrichment of fibrolytic taxa in high-altitude yak rumens, with associated differences in VFA production, methanogenesis, and starch metabolism among microbial communities [14,15]. These multifaceted adaptations collectively form the physiological basis for the yak’s efficient energy harvesting and prudent allocation strategies.

Currently, although numerous studies have partially uncovered the mechanisms underlying the high-altitude adaptation of yaks from perspectives such as host genomics and microbiome, a systematic review of the entire process of VFA generation, transport, absorption, and metabolism in the yak rumen is still lacking. Therefore, this paper aims to summarize recent advances in the mechanistic research on VFA generation, absorption, transport, and metabolism in the yak rumen. By systematically analyzing the adaptive characteristics of the VFA metabolic pathways in yaks, this review is expected to provide theoretical support for revealing the unique mechanisms of VFA generation and metabolism in the yak rumen under extreme environments.

## 2. Materials and Methods

This review was conducted following a systematic approach to ensure comprehensive and transparent literature selection. A systematic search of peer-reviewed articles was performed in the Web of Science, PubMed, and Scopus databases up to February 2026 using a Boolean combination of keywords: (“Yak” OR “*Bos grunniens*”) AND (“Rumen”) AND (“Volatile fatty acids” OR “VFA” OR “Short-chain fatty acids”) AND (“Production” OR “Fermentation” OR “Microbiota”) AND (“Transport” OR “Absorption” OR “Epithelium”) AND (“Metabolism” OR “Gluconeogenesis” OR “Lipogenesis”). The reference lists of all included articles were also manually screened. Studies were included if they were original research or reviews published in English, focused on yaks, and investigated any aspect of ruminal VFA dynamics. The initial search yielded approximately 320 records. After removing duplicates and screening titles and abstracts against the inclusion criteria (original research or reviews published in English focusing on yak rumen VFA dynamics), 170 full-text articles were assessed for eligibility. Ultimately, a total of 114 articles were included in this narrative synthesis, which integrated key findings thematically due to the heterogeneity of study designs.

## 3. Mechanisms of Rumen VFA Production

### 3.1. Primary Pathways of Rumen VFA Production

**Figure 1:**
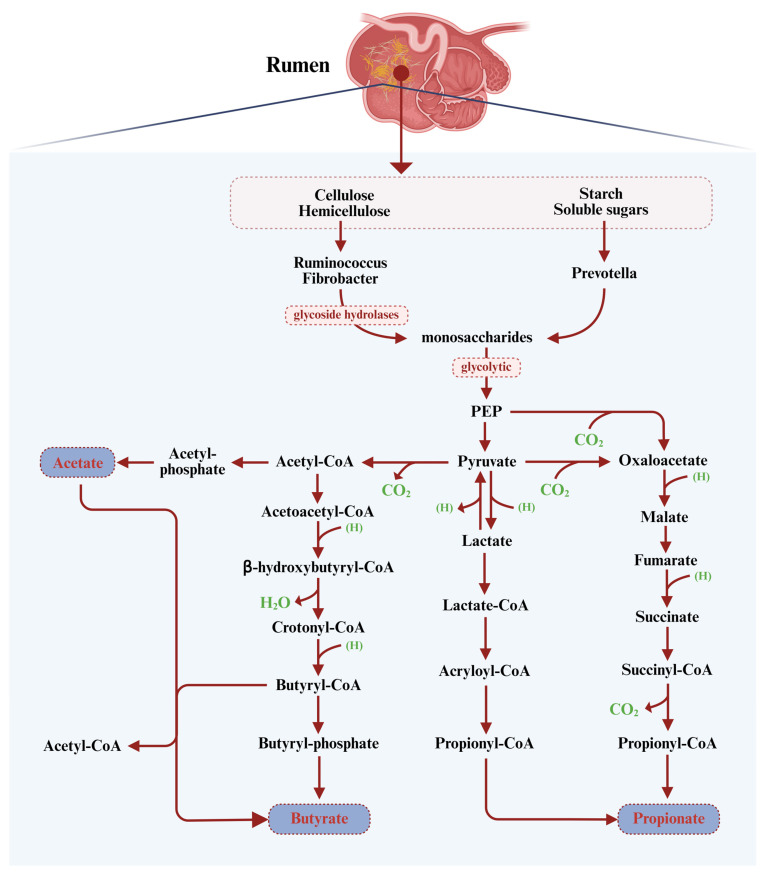
Mechanisms of volatile fatty acid production in the rumen. This schematic outlines the core metabolic routes through which dietary carbohydrates (structural and non-structural) are fermented by rumen microbiota to generate acetate (AA), propionate (PA), and butyrate (BA). Key intermediates and enzymes include phosphoenolpyruvic acid (PEP), pyruvate, acetyl-CoA, acetyl-CoA acetyltransferase (ACAT), and β-hydroxybutyryl-CoA dehydrogenase (BHBD). Hydrogen partial pressure is a principal factor modulating fermentation product proportions [18].

As illustrated in Figure 1, within the rumen, dietary carbohydrates—primarily cellulose, hemicellulose, starch, and soluble sugars—are anaerobically fermented by a complex microbial consortium, yielding VFAs via three core metabolic pathways [16]. From a ruminant nutrition perspective, carbohydrates are broadly classified into fiber (neutral detergent fiber, NDF) and non-fiber carbohydrates (NFCs), a distinction that fundamentally shapes ruminal fermentation patterns [17,18]. Fiber comprises slowly degradable structural components such as cellulose and hemicellulose, which are primarily fermented by fibrolytic bacteria into acetate as a major end-product, with the concurrent release of CO_2_ and H_2_ [19]. In contrast, the NFC fraction includes rapidly fermentable substrates such as starch, soluble sugars, and pectin. Although pectin is structurally a component of the plant cell wall, it is functionally classified as a non-fiber carbohydrate in detergent-based nutritional systems and follows a distinct, often faster, fermentation pathway [20]. Pectin is readily degraded by specific bacterial groups and typically yields acetate as the major product, but it can also contribute to propionate production due to its rapid fermentation [21]. Starch and soluble sugars are primarily fermented by amylolytic bacteria, with pyruvate being channeled into propionate via either the succinate or acrylate pathway. In the succinate pathway, the key intermediate succinate is further decarboxylated to propionate by specialist bacteria such as *Succiniclasticum* [13]. Butyrate is generated primarily via the acetate condensation pathway (catalyzed by enzymes such as acetyl-CoA acetyltransferase, ACAT, and β-hydroxybutyryl-CoA dehydrogenase, BHBD) or through direct lactate conversion [19].

A critical regulator of fermentation direction and VFA ratios is hydrogen partial pressure. Elevated H_2_ levels favor the production of more reduced products like propionate, thereby consuming hydrogen. Conversely, hydrogenotrophic methanogens lower H_2_ partial pressure, indirectly sustaining acetate-yielding pathways and influencing the final VFA profile [22]. In yaks, rumen content typically shows a high acetate proportion, likely due to their high-fiber, roughage-based diet and a distinctive microbial community. Comparative metagenomics suggests that the yak rumen microbiome possesses a unique carbohydrate-active enzyme (CAZyme) gene repertoire, particularly for cellulose/hemicellulose degradation, differing from lowland cattle and potentially contributing to this acetate-dominant pattern [23]. In summary, ruminal VFA production is a dynamic process orchestrated by a complex microbial network, with hydrogen partial pressure acting as a key metabolic regulator. The ultimate VFA yield and profile result from the multi-layered, dynamic interplay of diet, microbial community, host physiology, and environmental factors.

### 3.2. Key Factors Regulating Rumen VFA Production

#### 3.2.1. Dietary Structure and Nutritional Level

Diet composition is a primary determinant of rumen VFA profiles, exerting influence by altering the rumen microenvironment and the structure and function of resident microbial communities [24]. High-fiber diets promote acetate-dominated VFA synthesis by supplying abundant substrate for cellulolytic bacteria, stimulating their growth and activity, thereby increasing acetate yield and the acetate-to-propionate ratio [25]. In contrast, high-starch diets shift the microbial population towards starch utilizers, increasing propionate production [26]. Rapid acid generation from starch can lower rumen pH, inhibiting fibrolytic bacteria and altering fermentation patterns [27]. Thus, the relative abundance of dietary fiber versus starch shapes specific microbial consortia that in turn determine the major VFA outputs.

Beyond structure, dietary nutritional level significantly impacts VFA production. Energy and protein intake directly affect fermentation efficiency and VFA proportions [28]. High-energy diets elevate total VFA concentration and often increase the proportion of energetically efficient VFAs like propionate, enhancing feed conversion efficiency [12,29,30]. High-digestibility diets provide more fermentable substrate, supporting greater VFA output [31]. Notably, yaks often produce more VFAs than conventional cattle on identical diets [32,33], possibly due to a rumen microbiome with superior efficiency in capturing energy and protein from high-fiber forages [29]. Carbohydrate source also influences VFA profiles: a sudden switch to a high-concentrate diet rapidly increases total VFA concentration and shifts fermentation from an acetate- to a propionate-type, marked by falling acetate and rising propionate proportions [34]. Caution is warranted, as excessive starch can cause rumen pH to drop, raising the risk of subacute acidosis and disrupting VFA production. In Nubian goats, dietary molasses supplementation (2–6%) increased total VFA, with 4% and 6% levels significantly raising the proportion of propionate [35], underscoring the role of fermentable carbohydrates in modulating VFA composition. Therefore, precise manipulation of dietary nutrition and structure allows for targeted regulation of rumen fermentation, providing a rationale for optimizing production performance. In addition to the structure and nutritional level of the basal diet, the direct inclusion of functional feed additives represents a highly effective strategy for modulating rumen microbiota and VFA production profiles. Although studies specifically examining the effects of such additives in yaks remain scarce, extensive research in other ruminant species—particularly sheep and goats—has provided valuable mechanistic insights that can inform future yak-focused investigations. Table 1 summarizes representative studies in sheep and goats that have elucidated the effects of various feed additives on rumen microbiota and VFA production, offering a reference framework for designing analogous experiments in yaks.

#### 3.2.2. Rumen Microbial Community Structure

The rumen microbiome, often considered the host’s “second genome” [23], is dominated by the bacterial phyla Firmicutes and Bacteroidetes [44]. Firmicutes members, particularly families like Ruminococcaceae and Lachnospiraceae, are key fibrolytic players. Genera such as *Ruminococcus flavefaciens*, *R. albus*, and *Fibrobacter succinogenes* secrete a suite of carbohydrate-active enzymes (CAZymes)—endoglucanases, exoglucanases, β-glucosidases—that synergistically deconstruct plant cell wall polysaccharides into fermentable sugars [45]. F. succinogenes is especially notable for its unique cellulase system and strong adhesion to plant fiber [46]. Genomes of Ruminococcus and PseudoButyrivibrio are enriched with genes for cellulases (GH48) and hemicellulases (GH10), further aiding fiber breakdown [47]. Within Bacteroidetes, *Prevotella* is a major component (10–20% of bacteria) with broad carbohydrate metabolic capabilities. While not a primary degrader of crystalline cellulose, it degrades matrix components like pectin and xylan, facilitating access for cellulolytic specialists [48]. In Tianzhu white yaks, the dominant genera in both rumen and abomasum are *Rikenellaceae_RC9_gut_group* and *Christensenellaceae_R-7_group*, with functional prediction indicating that rumen microbes dominate amino acid metabolism—a configuration likely foundational for efficient digestion and VFA production [13]. While such predictions infer metabolic potential rather than actual in situ activity, this enrichment may reflect enhanced nitrogen cycling and microbial protein synthesis capacity within the rumen. Given that cellulolytic bacteria primarily utilize ammonia as their nitrogen source for growth [21], an increased capacity for nitrogen recycling could indirectly support fiber degradation by sustaining a more robust fibrolytic community. This, in turn, may contribute to the efficient VFA production observed in yaks, although direct experimental validation of this mechanistic link is still required.

The key functional microbes involved in ruminal fiber degradation and VFA production are summarized in Table 2. This table integrates foundational knowledge derived from diverse ruminant species with available evidence from yak-specific studies. Where yak data are lacking—particularly for certain butyrate- and propionate-producing taxa—information is extrapolated from other ruminant models (cattle, sheep, goats) to provide a comprehensive reference framework. Notably, recent metagenomic and cultivation-independent studies have confirmed the presence of many key fibrolytic and propionate-producing bacteria in the yak rumen, including Ruminococcus, Fibrobacter, Prevotella, and Succiniclasticum. However, direct functional validation of these taxa in yak-specific contexts remains an important avenue for future research.

#### 3.2.3. Host Physiological Stage

Ruminants dynamically adjust rumen fermentation and VFA output to meet the demands of different physiological stages, such as growth, gestation, and lactation [66,67]. In fattening stages, increased dietary protein enhances average daily gain [31], while higher energy intake raises ruminal propionate [29]. During lactation, heightened energy needs for milk synthesis shift fermentation towards greater energetic efficiency. Yaks in peak lactation maintain a high acetate output to support milk fat synthesis, and this increased VFA production is likely accompanied by adaptive changes in the rumen epithelium. Studies in other ruminant species, particularly cattle, have demonstrated that lactation induces rumen epithelial hyperplasia, with papillae surface area expanding by up to 32% under regulation by factors such as IGF-1, thereby enhancing VFA absorption capacity [68]. Although direct quantitative data on lactation-induced epithelial remodeling in yaks remain limited, the morphological plasticity of the yak rumen epithelium has been demonstrated in response to early nutritional interventions [69], suggesting that similar adaptive changes may occur during lactation, and transcriptomic evidence suggests that yaks possess the regulatory machinery (HIF-1 signaling pathway) to support such adaptations [70]. In alignment with this, research on different feeding methods for yak calves has shown significant impacts on growth performance, enzyme activity, and rumen microbial diversity, further emphasizing the critical role of early-life nutritional management [71]. Future studies are needed to quantify the extent of epithelial remodeling during lactation specifically in yaks and to confirm the involvement of IGF-1 and other regulatory factors.

#### 3.2.4. Other Environmental Factors

The chronic cold of high altitudes shapes yak feeding behavior and microbiome, thereby influencing VFA production and energy metabolism [27]. Cold stress increases thermogenic demands, leading to repartitioning of energy from production to maintenance and causing adaptive adjustments in VFA metabolism [72]. During the cold season when forage quality and quantity decline, yaks reduce feeding and rumination time while increasing locomotion, adopting a more dispersed grazing pattern [73]. Concurrently, ruminal concentrations of acetate, propionate, and total VFAs decrease, and the fermentation profile adjusts towards enhanced fiber degradation and energy capture efficiency—a strategy to optimize energy use during scarcity, albeit resulting in lower overall VFA output [74]. Blood metabolic profiling has revealed specific nutrient deficiencies in grazing yaks during the cold season, providing a physiological link between environmental stress and host metabolism [75]. The overarching importance of the gut microbiota for yak survival in this extreme environment has also been firmly established [76].

The yak rumen microbiome exhibits remarkable seasonal plasticity, adjusting its composition and function more robustly than in other bovids [46]. Seasonal shifts drive community succession: The relative abundance of Bacteroidetes and Tenericutes increases during the cold season, while Firmicutes and Synergistetes dominate in warmer periods [77]. *Prevotella* abundance rises significantly in winter when energy intake is limited [54]. Metagenomic analyses reveal the upregulation of lipid metabolism and glycan biosynthesis pathways in cold-season microbes [46], with succinate pathway gene abundance correlating positively with propionate production [78], suggesting *Prevotella* may boost propionate via this route. Dietary energy level is a key driver; *Prevotella* reaches its highest relative abundance under high-energy conditions [79], a pattern also observed in housed yaks with elevated concentrate feeding [29]. This highlights the microbiome’s nutritional plasticity. Collectively, through dynamic adjustments in community structure and metabolic function, the yak rumen microbiome helps the host navigate seasonal and nutritional fluctuations, ensuring relatively efficient VFA production. However, the subsequent fate of these VFAs—whether they are efficiently absorbed—hinges on the transport capacity of the rumen epithelium.

## 4. Transport Mechanisms of Rumen VFAs

### 4.1. Structural and Functional Basis of the Rumen Epithelium

The rumen epithelium (RE) is the principal interface for nutrient absorption and forms a crucial metabolic barrier [80,81]. It is organized into four layers: the stratum corneum (SC), stratum granulosum (SG), stratum spinosum (SS), and stratum basale (SB), which collectively perform barrier, absorptive, and metabolic functions [82]. The basal layer, adhering to the basement membrane, contains proliferative stem cells responsible for epithelial renewal and repair [83]. The spinous layer, rich in mitochondria, together with the basal layer, serves as a major site for VFA oxidation and ketogenesis [82]. The Hippo pathway effector YAP helps balance proliferation and differentiation in basal cells, which is essential for epithelial homeostasis and repair [84]. As cells migrate to the SG layer, mitochondrial numbers decline, but tight junction proteins (claudin-1, occludin, ZO-1) and desmosomes become denser, forming a selective barrier. This permits efficient transepithelial passage of VFAs, minerals, and vitamins while restricting harmful agents like endotoxins [85]. Barrier dysfunction can be mitigated by glutamine, which upregulates tight junction protein expression via p38 MAPK signaling and modulates apoptosis [86]. The outermost SC consists of keratinized, anucleated cells with wide intercellular spaces, providing a primary physical defense against microbes and abrasion [87].

Macroscopically, the papillated mucosa significantly increases the epithelial surface area, enhancing absorption and providing niches for microbial colonization, thus strengthening host–microbe interactions. A stage-specific, synergistic development exists between the mucosal microbiota and epithelial cells: early on, microbes help activate innate immunity for mucosal protection, while in maturity, fibrolytic bacteria interact with host metabolic genes to enhance nutrition acquisition [88,89]. Importantly, the RE is highly plastic. VFAs themselves can stimulate epithelial growth and differentiation via pathways like AMPK/PI3K, increasing papillae surface area and absorption capacity [55]. In yak calves, early supplementation with alfalfa hay and starter not only raises VFA production but also directly promotes papillae lengthening, widening, and rumen wall thickening, morphologically enhancing VFA absorption potential [69]. Studies in Tibetan sheep show dynamic expression of the monocarboxylate transporter 1 (MCT1) gene across phenological periods, optimizing short-chain fatty acid uptake [90]. Conversely, high-concentrate diets inducing subacute ruminal acidosis (SARA) disrupt tight junctions, increase permeability, and may trigger inflammation [86]. In summary, while the fundamental architecture and many regulatory mechanisms of the RE are conserved across ruminants, yaks exhibit both shared features and species-specific adaptations. However, substantial knowledge gaps remain regarding the molecular regulation of epithelial development, VFA transport, and barrier function specifically in yaks.

### 4.2. Principal Transport Mechanisms for Rumen VFAs

**Figure 2:**
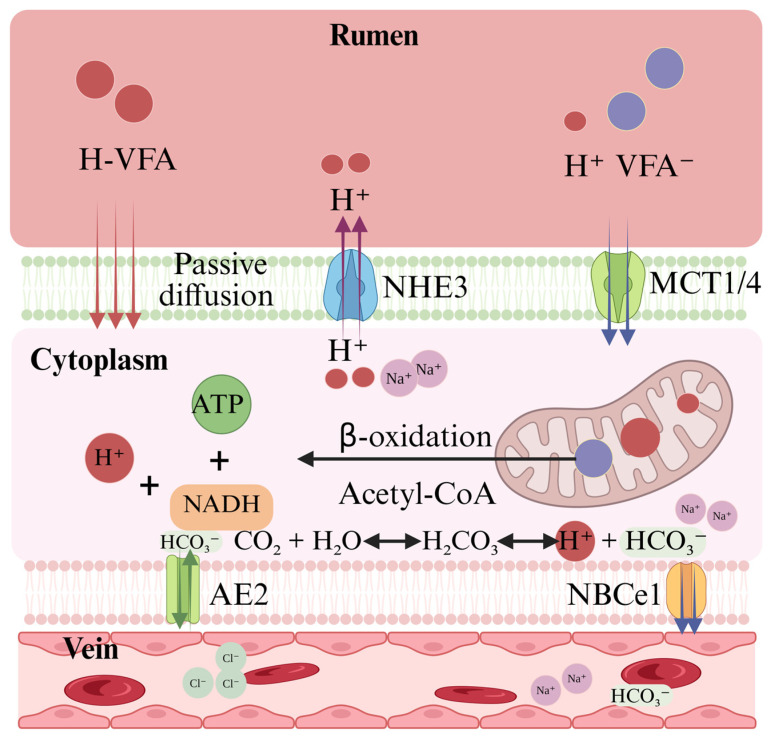
Transport pathways of VFA across the rumen epithelium. This diagram illustrates the three-level coordinated transport mechanism (using butyrate as an example). (1) Passive diffusion of the undissociated acid. (2) Carrier-mediated uptake facilitated by apical NHE3 and MCT1/4. (3) Metabolic pull driven by mitochondrial β-oxidation. Basolateral NBCe1 and AE2 help maintain pH homeostasis.

As illustrated in Figure 2, efficient VFA absorption across the rumen epithelium relies on a three-tiered, coordinated mechanism: passive diffusion, ion-coupled transport, and metabolic pull [91]. While this framework is well-established in cattle and goats, direct experimental validation in yaks remains limited. Nevertheless, based on the conserved nature of epithelial transport systems across ruminants, the following synthesis integrates knowledge from other species as a reference framework, highlighting available yak-specific evidence and critical knowledge gaps. Passive diffusion predominates at low rumen pH when VFAs are undissociated. However, at physiological pH (6.2–6.8), most VFAs exist in dissociated form (VFA^−^) and require carrier-mediated transport [92]. In conventional ruminants, transporter-mediated pathways account for approximately 70–75% of VFA absorption under normal pH conditions, with passive diffusion contributing the remaining 25–30% [92,93]. Although direct measurements in yaks are lacking, the conserved nature of these systems suggests a similar distribution, warranting experimental validation. Carrier-mediated transport of the remaining VFA^−^ fraction is facilitated by apical Na^+^/H^+^ exchanger 3 (NHE3), which exports H^+^ into the unstirred layer adjacent to the apical membrane [94,95]. This creates an acidic microclimate that promotes undissociated VFA formation and enables H^+^-coupled uptake via monocarboxylate transporters (MCT1 and MCT4) [94,95,96]. In yaks, however, the expression, localization, and functional activity of these transporters remain uncharacterized.

Metabolic pull represents the third critical component of VFA absorption. Upon entry, VFAs are rapidly oxidized in mitochondria, generating ATP and lowering intracellular concentration, which in turn drives further apical uptake [96]. Concurrently, H^+^ extruded via the respiratory chain (from β-oxidation-derived NADH) activates basolateral transporters—the sodium-bicarbonate cotransporter (NBCe1) and anion exchanger 2 (AE2)—which export metabolically generated HCO_3_^−^ into the blood, maintaining cytosolic pH. These pH-regulatory mechanisms have been characterized in detail in bovine and caprine rumen epithelium, but their molecular identity and regulation in yaks remain unexplored. While the tripartite model of VFA absorption—passive diffusion, carrier-mediated transport, and metabolic pull—provides a robust conceptual framework derived from studies in other ruminants, direct evidence for each component in yaks is either limited or entirely absent, this scarcity of species-specific data represents a fundamental obstacle to elucidating the unique adaptations underpinning the yak’s exceptional energy-harvesting capacity and demands urgent investigative attention. Transcriptomic analyses reveal changes in mRNA abundance of transporter genes [55,94], but mRNA levels do not always correlate with protein expression or functional activity due to post-transcriptional and post-translational regulation. Moreover, transporter capacity can be modulated by morphological changes in the rumen epithelium—increased papillae surface area, as observed in yaks during early nutritional interventions [69], enhances overall absorptive capacity even without changes in transporter expression per cell. Therefore, integrated assessments combining transcriptomics, proteomics, immunohistochemistry, and functional uptake studies are essential to fully understand VFA transport dynamics.

## 5. Metabolism of Rumen VFAs

### 5.1. Rumen Epithelial Metabolism

**Figure 3:**
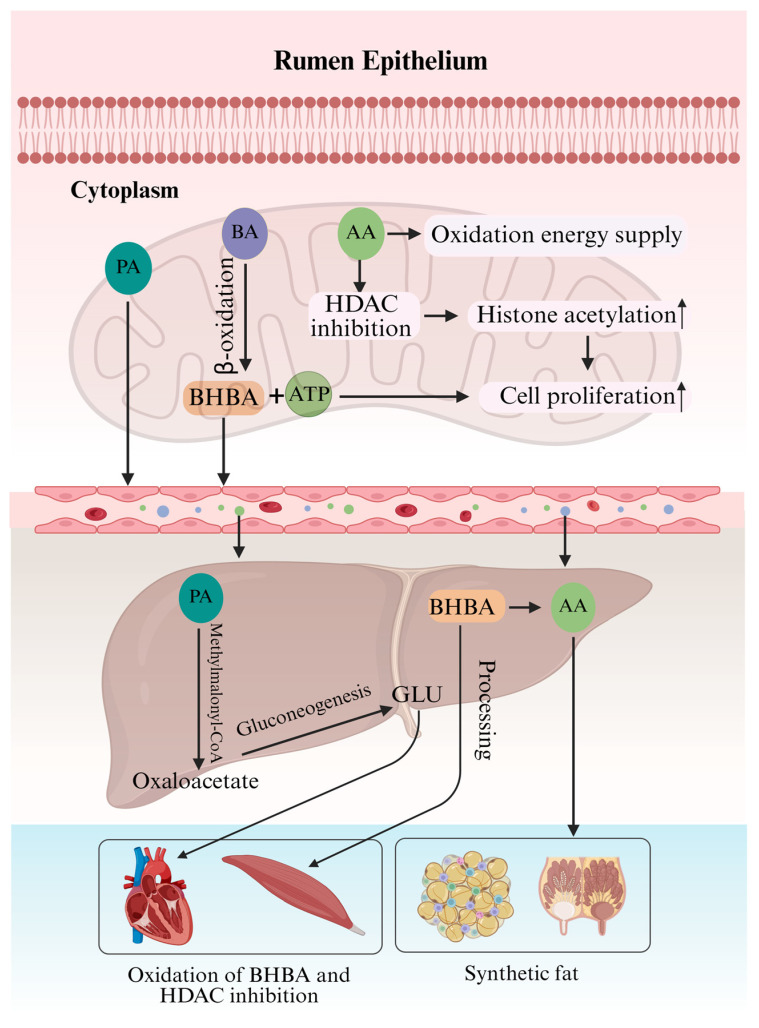
VFA metabolism in host tissues. A summary of the major metabolic destinies and allocation strategies of VFAs across key tissues. PA: propionate; BA: butyrate; AA: acetate; Acetyl-CoA: acetyl-CoA; OAA: oxaloacetate; BHBA: β-hydroxybutyrate; ACSS2: acyl-CoA synthetase short-chain family member 2; HDAC: histone deacetylase.

As summarized in Figure 3, the rumen epithelium is not merely an absorptive surface but also a primary metabolic processor of VFAs. Absorbed VFAs follow divergent metabolic routes within epithelial cells. In yaks, butyrate serves as the preferred energy substrate for ruminal epithelial cells, a characteristic conserved across ruminants. Transcriptomic analyses have revealed high expression of genes involved in butyrate metabolism and ketogenesis—including BDH1 (3-hydroxybutyrate dehydrogenase) and HMGCS2 (3-hydroxy-3-methylglutaryl-CoA synthase)—in the yak rumen epithelium, indicating active ketone body production [96]. Incomplete oxidation leads to the release of ketone bodies like β-hydroxybutyrate (BHBA) into the portal blood, supplying energy to peripheral tissues such as heart and muscle—thereby effectively transferring rumen-derived energy outward [97]. Acetate can also be oxidized but with lower priority and efficiency than butyrate [98]. Proteomic evidence further underscores the central role of epithelial energy metabolism in VFA absorption. A comparative study of normal versus growth-retarded yaks demonstrated that impaired mitochondrial ATP synthesis in rumen epithelial cells is associated with reduced VFA absorption efficiency, providing direct evidence for the functional importance of the metabolic pull mechanism in this species [96].

Beyond fueling, VFAs act as signaling molecules, directly modulating epithelial function through epigenetic mechanisms [99]. Acetate and butyrate, as histone deacetylase (HDAC) inhibitors, elevate histone acetylation, thereby opening chromatin and activating genes involved in proliferation, differentiation, and metabolism [100]. This epigenetic regulation has functional relevance in yaks: dietary supplementation with butyrate or butyrate-producing diets promotes rumen papillae development in yak calves, morphologically enhancing the epithelium’s absorptive capacity [71,101]. Thus, a dual mechanism operates: intracellular metabolic consumption drives continued VFA uptake by maintaining a favorable concentration gradient, while VFA-mediated signaling co-regulates epithelial development. Multi-omics analyses in yaks have revealed coordinated expression of genes involved in VFA transport, ketogenesis, and epithelial proliferation, supporting the integration of these mechanisms [55]. This synergy is a core strategy enabling yaks to maximize energy harvest from high-fiber diets.

### 5.2. The Central Role of the Liver

The liver is the principal site for propionate metabolism, converting it to glucose via the conserved methylmalonyl-CoA pathway—a critical gluconeogenic route in ruminants [102]. Propionyl-CoA, formed from propionate, is sequentially acted upon by propionyl-CoA carboxylase (PCC), methylmalonyl-CoA epimerase (MCEE), and methylmalonyl-CoA mutase (MUT) to yield succinyl-CoA, which enters the TCA cycle to produce oxaloacetate, a key gluconeogenic precursor [102]. In yaks, the importance of hepatic gluconeogenesis is amplified by their low-starch, high-fiber diet and the extreme seasonal fluctuations in energy supply. Transcriptomic profiling of yak liver has revealed high constitutive expression of key gluconeogenic enzymes, including PCK1 (phosphoenolpyruvate carboxykinase) and G6PC (glucose-6-phosphatase), suggesting an enhanced capacity for propionate-to-glucose conversion [103]. This adaptation is particularly critical during the cold season, when energy demands increase while dietary energy density declines. This pathway supplies essential glucose precursors, which is particularly vital for yaks consuming low-starch, high-fiber diets. Acetate and butyrate-derived BHBA are metabolized differently. The liver is not a major oxidation site for these but rather a processing and distribution center. Much of the acetate bypasses hepatic metabolism to enter systemic circulation, while ketone bodies from ruminal butyrate oxidation, along with hepatic ketogenesis, are released into peripheral blood [104]. The liver itself can also produce and release acetate into circulation [105]. Metabolomic studies in yaks provide direct evidence for this carbon partitioning strategy. During the cold season, when energy intake is limited, yaks exhibit elevated circulating levels of ketone bodies (BHBA and acetoacetate) alongside maintained blood glucose concentrations, indicating that hepatic ketogenesis and gluconeogenesis operate in parallel [75]. This metabolic flexibility enables tissue-specific energy allocation: the liver prioritizes propionate for gluconeogenesis to preserve glucose homeostasis, while directing acetate and ketone bodies—two key alternative fuels—to peripheral tissues [106]. When propionate influx exceeds gluconeogenic capacity, the surplus can be converted to acetate or channeled into lipogenesis [107], providing metabolic flexibility that prevents toxicity and optimizes energy partitioning. This buffering capacity is particularly important given the pronounced seasonal variation in both energy intake and expenditure. Transcriptomic analyses of yak liver across seasons have revealed dynamic regulation of genes involved in lipid metabolism and ketogenesis, supporting the liver’s role as a metabolic buffer that adapts to fluctuating energy availability [103]. For yaks facing pronounced seasonal energy intake fluctuations, the liver’s dynamic regulation of VFA metabolic flux is a key adaptive trait, acting as a metabolic buffer crucial for energy efficiency and homeostasis in the variable alpine environment.

### 5.3. Energy and Anabolic Metabolism in Peripheral Tissues

Acetate is the primary carbon source for de novo lipogenesis in ruminants [108]. In mammary and adipose tissue, acetate is activated to acetyl-CoA by acyl-CoA synthetase short-chain family member 2 (ACSS2), fueling cytoplasmic fatty acid synthesis [109]. The typically low activity of ATP-citrate lyase (ACLY) in ruminants underscores acetate’s central role. High ACSS2 expression in yak mammary epithelium correlates with higher proportions of medium- and short-chain fatty acids in milk fat, a direct outcome of acetate utilization [110]. Beyond substrate supply, acetate can modulate lipid metabolism via AMPK signaling [103]. Ketone bodies serve as efficient oxidative fuels for tissues like cardiac and skeletal muscle, especially under glucose-sparing conditions [106]. BHBA also functions as an epigenetic regulator by inhibiting HDACs [111]. It is noteworthy that acetic acid and ketone bodies do not function independently; rather, they work synergistically to optimize energy allocation. Specifically, acetic acid is primarily utilized for fat synthesis, promoting energy storage, while ketone bodies serve as efficient immediate energy sources, ensuring the proper functioning of tissues and organs [13]. During lactation, mammary demand for acetate surges for milk fat synthesis, and ketone bodies offer supplemental energy [110]. Recent lipidomics has linked specific triglycerides in yak milk to volatile flavor compounds, with lipid hydrolysis and derivatization playing key roles in flavor development [112]. During fasting or exercise, lipolysis increases ketogenesis for systemic energy, while acetate utilization may decline [103]. This adaptable synergy enables yaks to meet diverse physiological demands under varying environmental and metabolic conditions.

### 5.4. The Microbial-Host Synergistic Metabolic Network

Yak VFA metabolism exemplifies a co-evolved, efficient energy extraction and utilization network shaped by the plateau’s energy constraints. During cold-season scarcity, the enrichment of key taxa like *Prevotella* and upregulation of their succinate pathway genes increase propionate yield, thereby supplying more gluconeogenic precursor [77]. The host complements this by optimizing absorption and metabolism: the rumen epithelium highly expresses ketogenic genes to efficiently convert butyrate into peripheral energy (ketone bodies) [77], while the liver maintains robust gluconeogenic capacity from propionate [103]. Critically, this system is bidirectional. Host physiology (via diet and internal milieu) reshapes the microbiome, while microbial VFAs act as signals that continuously fine-tune host gene expression and metabolism [113]. This integrated network, spanning microbial fermentation to multi-tissue host utilization, constitutes a core metabolic foundation for yak survival and reproduction in the energy-limited highlands.

## 6. Research Gaps and Future Perspectives

Despite considerable progress in characterizing ruminal VFA metabolism in yaks, we still lack a detailed molecular understanding of how these microbial end-products are sensed, transported, and utilized by yak host tissues. Current evidence on yak rumen VFA metabolism remains largely correlative, often linking changes in microbial taxa to shifts in fermentation parameters without establishing causal mechanisms. Additionally, the activation patterns of VFA signaling pathways that have been clarified in lowland ruminants in the yak rumen epithelium at the cell-type-specific level have not been studied, and their functional roles in yak VFA metabolism cannot be directly inferred from lowland ruminant results. Another significant limitation in current yak research is the lack of validated, species-specific antibodies for key VFA transporters (MCT1, MCT4, NHE3, AE2, NBCe1). Although transcriptomic studies have demonstrated dietary- and season-induced changes in transporter mRNA abundance in yaks [29,55], whether these transcriptional changes translate to altered protein expression or subcellular localization remains unknown due to the absence of reliable immunological tools. Furthermore, functional studies using specific transport inhibitors—such as AR-C155858, a potent inhibitor of MCT1 and MCT2 with minimal affinity for MCT4—have been instrumental in dissecting the contribution of individual monocarboxylate transporters to VFA absorption in bovine and other species. To date, no such inhibitor-based investigations have been performed in yak rumen epithelium.

To address these gaps, future research should prioritize the development and validation of yak-specific antibodies and the application of functional transport assays. Integrated approaches combining transcriptomics, proteomics, and immunohistochemistry with Ussing chamber studies using specific inhibitors (AR-C155858 for MCT1, selective NHE3 inhibitors) would enable direct assessment of transporter activities and their regulatory mechanisms in yak rumen epithelium. Additionally, single-cell RNA sequencing could resolve the cellular heterogeneity of the yak rumen epithelium, identifying specialized cell populations responsible for VFA transport and metabolism. Such studies across different physiological states (lactation, cold stress, growth) and seasons would elucidate how yaks dynamically regulate VFA absorption and metabolism to adapt to extreme environmental fluctuations. Ultimately, these mechanistic insights could inform nutritional strategies to enhance rumen efficiency not only in yaks but also in other ruminants facing environmental stress.

## 7. Conclusions

The adaptation of yaks to the energy-limited environment of the Qinghai–Tibet Plateau is closely linked to the efficiency with which rumen-derived volatile fatty acids are produced, absorbed, and metabolized. Seasonal plasticity of the rumen microbiome, combined with specialized epithelial absorption and coordinated metabolic utilization of VFAs across tissues, underpins this adaptive strategy. Together, these processes enable yaks to maintain productivity under harsh environmental conditions. Nevertheless, as current evidence remains largely correlative, further integrative studies are required to clarify the mechanisms linking rumen function, host metabolism, and productive traits in this species. Given their remarkable capacity to maintain productivity under extreme nutritional and environmental stress, yaks offer a unique model system for understanding the mechanisms of rumen efficiency that could inform strategies to enhance livestock resilience and productivity worldwide, particularly in the face of climate change and increasing feed scarcity.

## Figures and Tables

**Table 1:** Effects of different feed additives on rumen microbiota and VFA production.

Additive Category	Specific Additive	Test Animal	Key Effects on Rumen Microbiota	Key Effects on VFA Production	References
Plant By-products	*Lycium barbarum* (Wolfberry) Residue	Tan sheep	*Prevotella*, *Ruminococcus*, *Butyrivibrio* ↑	Total VFA ↑	[36]
Plant Extracts	*Astragalus membranaceus* Root	Goats	Community diversity ↑, *Oscillospirales* ↑	Total VFA ↑	[37]
Plant By-products	Residual Black Wolfberry Fruit	Duolang sheep	*Prevotella*, *NK4A214 group* ↑	Total VFAs, acetate, propionate, butyrate ↑	[38]
Plant By-products	*Lycium barbarum* Branches and Leaves	Hu sheep	—	Propionate, valerate ↑	[39]
Fermented Feeds	Fermented Rice Husk Powder	Hu sheep	*Rikenellaceae RC9* gut group ↓; *Succinivibrio* ↑	Total VFA, butyrate, valerate ↑	[40]
Fermented Feeds	Ensiled Rice Straw	Hu sheep	Bacterial diversity ↑, *Methanobrevibacter* ↓	Acetate, organic acids in vitro ↑	[41]
Probiotics	*Saccharomyces cerevisiae* (Live Yeast)	Hu Lambs	*Butyrivibrio*, *Pseudobutyrivibrio* ↑	Total VFA, acetate, propionate ↑	[42]
Probiotics	*Saccharomyces cerevisiae*	Growing Goats	—	Propionate, total VFA ↑	[43]

Note: ↑ indicates an increase; ↓ indicates a decrease. Dashes (—) indicate no reported effects or not applicable. In vitro denotes results from in vitro fermentation studies.

**Table 2:** Key functional microorganisms and their roles in VFA metabolism.

Microbial Taxa	Primary Substrates	Primary VFA Products	References
Fibrolytic Bacteria			
*Ruminococcus flavefaciens*	Cellulose, Hemicellulose	Acetate, H_2_, Formate	[47]
*Ruminococcus albus*	Cellulose, Hemicellulose	A, Formate, Ethanol	[47]
*Fibrobacter succinogenes*	Crystalline cellulose	Acetate, Succinate	[46]
Butyrate-Producing Bacteria			
*Butyrivibrio fibrisolvens*	Xylan, pectin, soluble sugars, cellulose	Butyrate, Formate, Lactate	[49]
*Pseudobutyrivibrio xylanivorans*	Xylan, hemicellulose	Butyrate	[50]
*Butyrivibrio proteoclasticus*	Hemicellulose, pectin, proteins	Butyrate	[49]
*Roseburia* spp.	Soluble sugars, starch, xylan	Butyrate	[51]
*Agathobacter ruminis* (*formerly Eubacterium* spp.)	Glucose, cellobiose, soluble sugars	Butyrate, Acetate, Lactate, H_2_	[52]
*Agathobacter rectalis* (*formerly Eubacterium rectale*)	Soluble sugars, starch	Butyrate, Acetate, Lactate, H_2_	[52]
*Oribacterium* spp.	Soluble sugars	Butyrate, Acetate	[53]
Propionate-Producing Bacteria			
*Prevotella ruminicola*	Starch, soluble sugars, hemicellulose, pectin	Acetate, Propionate, Succinate	[23,54]
*Prevotella bryantii*	Starch, xylan	Propionate, Acetate	[23]
*Selenomonas ruminantium*	Lactate, soluble sugars, starch, glycerol	Propionate, Acetate, Lactate	[55,56]
*Succiniclasticum ruminis*	Succinate	Propionate	[57]
*Ruminobacter amylophilus*	Starch, maltodextrins	Acetate, Propionate, Succinate	[58]
*Succinivibrio dextrinosolvens*	Starch, dextrin, pectin	Succinate, Acetate	[56,59]
Specialized Functional Bacteria			
*Anaerovibrio lipolytica*	Lipids, glycerol	Propionate, Acetate, Succinate	[60]
*Treponema bryantii*	Soluble sugars, xylan	Acetate, Formate, Succinate	[61]
*Wolinella succinogenes*	Formate, H_2_, fumarate	Succinate	[62]
*Victivallis vadensis*	Cellobiose, glucose, soluble sugars	Acetate, Ethanol, H_2_	[63]
*Sharpea azabuensis*	Soluble sugars	Lactate, Acetate, Ethanol	[64]
*Kandleria vitulina*	Soluble sugars	Lactate, Acetate	[65]
Functional Groups			
Lachnospiraceae family	Diverse carbohydrates	Butyrate, Acetate	[46]
Ruminococcaceae family	Cellulose, hemicellulose	Acetate, Butyrate	[27,46]

## Data Availability

No new data were created or analyzed in this study. Data sharing is not applicable to this article.

## Abbreviations

The following abbreviations are used in this manuscript:

VFAs	Volatile Fatty Acids
SCFA	Short-chain fatty acids
AA	Acetate
PA	Propionate
BA	Butyrate
BHBA	β-Hydroxybutyrate
PEP	Phosphoenolpyruvate
NDF	Neutral detergent fiber
NFCs	Non-fiber carbohydrates
TCA cycle	Tricarboxylic Acid Cycle
CAZymes	Carbohydrate-Active Enzymes
ACAT	Acetyl-CoA Acetyltransferase
BHBD	β-Hydroxybutyryl-CoA Dehydrogenase
HIF-1	Hypoxia-Inducible Factor-1
ACSS2	Acyl-CoA Synthetase Short-Chain Family Member 2
HDAC	Histone Deacetylase
MCT1/4	Monocarboxylate Transporter 1/4
NHE3	Na^+^/H^+^ Exchanger 3
NBCe1	Sodium-Bicarbonate Cotransporter 1
AE2	Anion Exchanger 2
PCK1	Phosphoenolpyruvate carboxykinase
G6PC	Glucose-6-phosphatase
PCC	Propionyl-CoA carboxylase
MCEE	Methylmalonyl-CoA epimerase
MUT	Methylmalonyl-CoA mutase
IGF-1	Insulin-like Growth Factor-1
AMPK	AMP-activated Protein Kinase
PI3K	Phosphoinositide 3-Kinase
YAP	Yes-associated protein
RE	Rumen Epithelium
SC	Stratum Corneum
SG	Stratum Granulosum
SS	Stratum Spinosum
SB	Stratum Basale
SARA	Subacute Ruminal Acidosis
peNDF	physically effective Neutral Detergent Fiber
RDS	Rumen Degradable Starch
GH	Glycoside Hydrolase
